# A deep database of medical abbreviations and acronyms for natural language processing

**DOI:** 10.1038/s41597-021-00929-4

**Published:** 2021-06-02

**Authors:** Lisa Grossman Liu, Raymond H. Grossman, Elliot G. Mitchell, Chunhua Weng, Karthik Natarajan, George Hripcsak, David K. Vawdrey

**Affiliations:** 1 Department of Biomedical Informatics, Columbia University, New York, NY USA; 2 Kensho Technologies, LLC, Cambridge, MA USA; 3 Steele Institute for Health Innovation, Geisinger, Danville, PA USA

**Keywords:** Health care, Medical research

## Abstract

The recognition, disambiguation, and expansion of medical abbreviations and acronyms is of upmost importance to prevent medically-dangerous misinterpretation in natural language processing. To support recognition, disambiguation, and expansion, we present the Medical Abbreviation and Acronym Meta-Inventory, a deep database of medical abbreviations. A systematic harmonization of eight source inventories across multiple healthcare specialties and settings identified 104,057 abbreviations with 170,426 corresponding senses. Automated cross-mapping of synonymous records using state-of-the-art machine learning reduced redundancy, which simplifies future application. Additional features include semi-automated quality control to remove errors. The Meta-Inventory demonstrated high completeness or *coverage* of abbreviations and senses in new clinical text, a substantial improvement over the next largest repository (6–14% increase in abbreviation coverage; 28–52% increase in sense coverage). To our knowledge, the Meta-Inventory is the most complete compilation of medical abbreviations and acronyms in American English to-date. The multiple sources and high coverage support application in varied specialties and settings. This allows for cross-institutional natural language processing, which previous inventories did not support. The Meta-Inventory is available at https://bit.ly/github-clinical-abbreviations.

## Abstract (summary)

**Table:** 

Measurement(s)	Controlled Vocabulary • Linguistic Form
Technology Type(s)	digital curation • data combination
Sample Characteristic - Location	United States of America

Machine-accessible metadata file describing the reported data: 10.6084/m9.figshare.14068949

## Background & Summary

Natural language processing (NLP) is becoming essential to health and healthcare^1,2^. NLP translates free text and speech into standardized data^3^, which can help clinicians make decisions^4^, predict health outcomes^5^, prevent adverse events^6^, and improve quality-of-care^1,2^. In the past few years, artificial intelligence breakthroughs using pre-trained transformer architectures have revolutionized NLP^7^. These breakthroughs have empowered researchers to build generalizable language models and apply them to achieve superior accuracy on subsequent downstream tasks^8^. Since then, pre-trained transformer architectures have become mainstream for language tasks involving contextual long-distance dependencies, and have been incorporated into commercial services such as Google Search^9^ and Amazon Alexa^10^.

Despite these recent advancements, clinical abbreviations and acronyms (hereafter, ‘abbreviations’) persistently impede NLP performance and practical application in health and healthcare^11–19^. Abbreviations constitute 30–50% of the words in clinical text, such as doctor’s notes^20^, compared to <1% in general text, such as news media^21^. As such, recognizing, disambiguating, and expanding abbreviations is central to clinical NLP, and even small advancements would improve performance and practical application^11–19^. Furthermore, recognizing, disambiguating, and expanding abbreviations can help physicians, nurses, caregivers, and patients understand them, which studies have shown prevents medically-dangerous misinterpretation^22–26^.

Recognition, disambiguation, and expansion of abbreviations relies on *sense inventories*, defined as databases of abbreviations and their meanings or *senses*. Large sense inventories can be publicly obtained online (e.g., Unified Medical Language System [UMLS], nlm.nih.gov/research/umls) but they can be incomplete^13,19,27,28^, because they were generated using biological research corpora such as research papers, not clinical corpora such as electronic health records^13,29^. Due to this limitation, several institutions have engineered their own smaller, more clinically-oriented sense inventories^30–36^. These inventories are sufficient for institution-specific tasks, but have been inadequate for cross-institutional (interoperable) tasks, because abbreviations vary substantially based on medical specialty and setting^23–26^. Sadly, creating inventories at every US healthcare institution is not feasible, especially without fully automated methods which do not exist.

*Deep data* refers to high-quality, complete, and relevant data with an internal structure that may be large-scale^37,38^. A *deep* sense inventory that is high-quality, complete, relevant, and non-redundant could solve the problems of interoperability and generalizability. Generating such an inventory would require extraction, collation, and organization of numerous source inventories. Collating a deep sense inventory is challenged by two major obstacles. First, errors have been recognized in several sources^20,39^, necessitating quality control to remedy them. Second, because abbreviations vary based on specialty and setting, numerous individual sense inventories from different specialties and settings are needed. Using numerous inventories increases the likelihood of considerable redundancy, necessitating cross-mapping (internal structure) to remove redundancy and simplify future application. This cross-mapping is prohibitive to perform manually due to the combinatorial nature of the problem, as the number of comparisons increases exponentially with the number of records.

Here, we present a deep database of medical abbreviations and acronyms, which harmonizes multiple source sense inventories from varied corpora, medical specialties, and medical settings into one *Meta-Inventory*. The Meta-Inventory has two major features that address the challenges stated above: [1] semi-automated quality control using heuristics to identify errors and improve reliability, and [2] automated cross-mapping of synonyms using state-of-the-art machine learning to remove redundancy and simplify future downstream tasks.

Additional features include lexical normalization of non-standard to standard text, assignment of unique identifiers to streamline maintenance and use, and transparency to prevent information loss secondary to harmonization. As NLP is increasingly used in healthcare, the Meta-Inventory will be an essential resource to better recognize, disambiguate, and expand medical abbreviations across multiple institutions, specialties, and settings.

## Methods

### Data sources

We included inventories from government sources, online repositories, and peer-reviewed scientific literature. Government sources included the UMLS Lexical Resource for Abbreviations and Acronyms (UMLS-LRABR)^40^, and online repositories included Another Database of Abbreviations in Medline (ADAM)^41^. Since UMLS-LRABR and ADAM were generated using biological research corpora, we augmented these data sources with more clinically-oriented inventories, including Berman’s abbreviations^42^, Wikipedia^43^, and inventories from Vanderbilt University Medical Center^44^ and Columbia University Irving Medical Center^45^. The clinically-oriented inventories were generated from clinical corpora using various manual and semi-automated methods. Table 1 describes every sense inventory in the Meta-Inventory. We only included sense inventories with no copyright for any use, without restrictions for any use (e.g., CC0), or any use with attribution (e.g., CC BY). Sources with copyright restrictions (e.g., All Acronyms) or with 100% overlap were not included.

**Table 1:** Source Sense Inventories.

Source	Description	Underlying Corpus	Medical Specialty	Last Updated	Records
UMLS-LRABR^40^	Unified Medical Language System Lexical Resource for Abbreviations and Acronyms	Biomedical research	Multiple	2019	294484
ADAM^41^	Another Database of Abbreviations in Medline	Biomedical research	Multiple	2007	94657
Berman^42^	Manually-curated general pathology abbreviations	Clinical notes	Pathology	2004	12087
Wikipedia^43^	Publicly-curated list of medical and clinical trial abbreviations	Clinical notes	Multiple	2018	2952
Vanderbilt1^44^	Semi-automatically derived from the medical record	Sign-out notes	Medicine	2013	2414
Vanderbilt2^44^	Semi-automatically derived from the medical record	Discharge notes	Medicine	2013	2090
Stetson^45^	Manually-curated from the general medical record	Sign-out notes	Medicine	2002	765
Columbia	Manually-curated from the obstetric medical record	Clinical notes	Obstetrics	2018	219

### Data harmonization

The database structure was inspired by the UMLS Metathesaurus^46^, a federally-maintained repository of biomedical terms organized by concept^47,48^. To achieve concept-orientedness, the UMLS Metathesaurus cross-maps *synonyms*, or individual terms related to the same concept. The UMLS Metathesaurus offers a stable and well-known framework to guide cross-mapping and ensure full *source transparency*, or link-back to the original sources^49^. Moreover, it provides standard names, definitions, and formats for certain data fields, which we hope will give researchers familiar with the UMLS Metathesaurus an intuition for the Meta-Inventory.

**Fig. 1:**
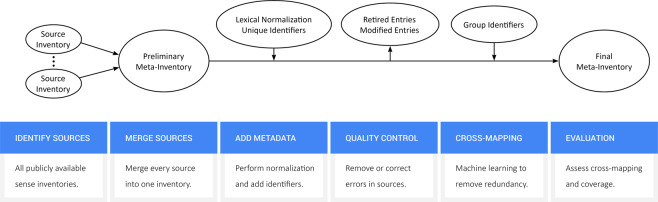
Overview of Data Harmonization.

We included the following data fields found in each source: [A] *short form*, or the abbreviation (e.g., “MS”); [B] *long form*, or the spelled-out version of the abbreviation (e.g., “Multiple Sclerosis”); [C] *source*, or the name of the source inventory. Each individual record (row) represents a single abbreviation (short form) and corresponding sense (long form). Then, we created the following new data fields: [D] *normalized short form*, or a lexically normalized version of each short form, intended to reduce linguistic variation; [E] *normalized long form*, or a lexically normalized version of each long form, intended to reduce linguistic variation; [F] *unique identifiers* for each individual record, each unique short form, and each unique long form, intended to facilitate future database maintenance and use; [G] *group identifiers* for each group of synonymous (i.e., cross-mapped) records, intended to reduce redundancy. We detail each new data field, its purpose, and its creation below. Figure 1 provides an overview of the data harmonization process.

### Lexical normalization

Linguistic variation can degrade the effectiveness and increase the complexity of NLP. Lexical normalization can reduce linguistic variation and thereby improve the recognition and identification of abbreviations and their senses in clinical text. We performed short form normalization using Clinical Abbreviation Disambiguation and Recognition (CARD; https://sbmi.uth.edu/ccb/resources/abbreviation.htm), an open-source framework for abbreviation identification and normalization^30^. Briefly, CARD converted short forms to lowercase, stripped leading and trailing whitespace, removed periods, and standardized remaining punctuation to an underscore.

We performed long form normalization using the UMLS Lexical Variation Generation (UMLS-LVG; https://nlm.nih.gov/research/umls) version 2019AB, an open-source toolset for transforming clinical text into a single canonical (i.e., normalized) form^50^. We modified the standard UMLS-LVG normalization flow options to avoid alphabetical sorting. The final modified flow was q0:g:rs:o:t:l:B:Ct:q7:q8. Briefly, this flow standardized the character encoding (q0, q7, and q8), removed genitives (g), stripped plural forms (rs), replaced punctuation (o), removed stop words (t), converted to lowercase (l), uninflected (B), and identified synonyms (Ct). In cases where UMLS-LVG could not perform lexical normalization, such as chemical names, we recorded “null” values.

### Unique identifiers

We assigned non-semantic unique identifiers^47,48^ to facilitate database maintenance and future use, specifically quality control. We formatted each identifier as a six-digit number prefaced with “R” for individual records (e.g., R000001, R000002, …), “S” for unique short forms (e.g., S000001, S000002, …), and “L” for unique long forms (e.g., L000001, L000002, …). To preserve source transparency, we assigned record unique identifiers in the original order of the source.

### Cross-mapping

We cross-mapped synonymous records using an automated pipeline to reduce redundancy. To automate cross-mapping, we constructed and explored the performance of two machine learning models. These models were employed because previous approaches using MetaMap were inadequate^20,48^, as MetaMap only identifies 30% of the Meta-Inventory. We used a 3-step approach:

1. *initial filtering* to identify potentially synonymous records and generate training data;
2. *construction and evaluation* of potential models and ensembles for cross-mapping;
3. *assign group identifiers* to records cross-mapped using the best-performing ensemble.

We selected pairwise comparison as the basis for our modeling pipeline^51^. While many deduplication problems cannot be tackled easily with pairwise comparison due to the polynomial nature of combination, pairwise comparison is appropriate for this problem because only potential pairs within the same short form were considered. In other words, we paired records with: [A] the same short form, and [B] long forms with the same meaning. This was important to streamline future application to abbreviation disambiguation and expansion. The target for each pair consisted of a binary value indicating whether or not the long forms were synonyms.

#### Initial filtering

The standard Levenshtein distance ratio^49^, which measures string similarity, was computed between every long form. Pairs of long forms where ratio >0.8 with an equivalent short form were identified as potential positives (i.e., synonyms), and pairs where ratio >0.8 without an equivalent short form were identified as potential pertinent negatives (i.e., not synonyms, but similar). A clinician manually annotated 1% samples of each as positive or negative (~10,000 pairs). We supplemented this training data with manually-identified pertinent positives and negatives, lists of medical synonyms^37^, and synonymous relationships pre-recorded in the UMLS-LRABR.

#### Construction and evaluation

The data preparation for the modeling pipeline consisted of three steps. First, we normalized the textual data by replacing unusual textual features such as roman numerals, decimals, and common ions with their long-form text. Subsequently, we identified potential pairs in the Meta-Inventory. Pairs that had a partial Levenshtein distance ratio of 0.5 or above were considered. We identified 3 million potential pairs. Finally, we calculated string similarity metrics to use as features, including: [1] Levenshtein distance, [2] partial Levenshtein distance, [3] token-sort Levenshtein distance, [4] token-set Levenshtein distance, and [5] numeric similarity.

Then, we passed these features to a feedforward dense neural network (baseline), a gradient boosted model (LightGBM)^52^, and a transformer model (BERT)^7^ for training and evaluation. The baseline and LightGBM only used the features from the data preparation. The transformer model additionally passed the normalized text for each pair through BERT to generate a text embedding as additional features, and then used these features in conjunction with the preprocessed features to generate a prediction through a feedforward model head. We conducted a sensitivity analysis around the version used (BERT^7^ vs. Clinical BERT^53^), and BERT outperformed Clinical BERT. We cross-validated the models in a K-fold manner on the clinician-labeled training data.

The LightGBM and BERT pipelines performed comparably on the clinician-labeled training data (Table 2). We posit that BERT did not outperform LightGBM because LightGBM excels at finding decision boundaries in small-data problems, whereas dense neural networks do not. Additionally, these data samples lack context such as nearby clauses or sentences. An ensemble of the two models did not significantly improve the F1 score. It is important to note that these scores are calculated on the clinician-labeled training data which consists primarily of “difficult” pairs, that is, positives and pertinent negatives close to the class boundary of the problem (described above in the “Initial filtering” section). On the complete set of potential pairs, which included negatives that were very dissimilar and positives that were nearly identical, the scores increased significantly (>0.98).

**Table 2:** Performance of Cross-Mapping Models on Clinician-Labeled Data*.

Model	Precision	Recall	F1 Score
Baseline	0.788	0.759	0.773
LightGBM	0.813	0.785	0.799
BERT Architecture	0.815	0.772	0.793
Ensemble	0.828	0.801	0.814

*Scores calculated using the mean predictions of 3 runs with different random seeds.

#### Assign group identifiers

Using the best-performing model, the LightGBM trained on string similarity metrics, we cross-mapped synonymous records. Each group of synonymous records received a unique identifier, prefaced with “G” for group (e.g., G000001, G000002, …). Records without any synonyms were assigned their own group.

### Source transparency

The Meta-Inventory should represent its sources transparently, without any information loss due to abstraction or manipulation, to preserve attributes of each record^49^. Occasionally, the source sense inventories contained auxiliary data fields unique to that source. To preserve transparency, we created a version of the Meta-Inventory with every auxiliary data field. Examples of auxiliary fields include: [A] *type*, abbreviation or acronym (original source: UMLS-LRABR); [B] *preferred short form*, or the preferred lexical version of each abbreviation (original source: ADAM); [C] *frequency*, or how often that abbreviation takes that meaning in the given clinical corpora (original source: Vanderbilt).

## Data Records

The latest release of the Meta-Inventory is archived on Zenodo (https://zenodo.org/record/4266962)^54^, and subsequent releases will also be archived there. The latest release can also be downloaded from the corresponding GitHub repository (https://bit.ly/github-clinical-abbreviations). In addition to the Meta-Inventory, the Zenodo and GitHub repositories contain the open source license (Apache License Version 2.0), the version with auxiliary data fields, the source inventories, the training datasets, the entire code, and the documentation of modified or retired records. The data dictionary (Table 3) contains the documentation of the data fields and sample values.

**Table 3:** Data Dictionary.

Data Field		Name		Description		Example	
GroupID		Group Unique Identifier		Identifies a group of synonymous records		G169326	
RecordID		Record Unique Identifier		Identifies each record (one per record)		R349343	
SF		Short Form		Abbreviated version of an abbreviation		O.C.	
SFUI		Short Form Unique Identifier		Identifies a unique short form		S050750	
NormSF		Normalized Short Form		Lexically normalized version of the short form		oc	
LF		Long Form		Spelled-out version of an abbreviation		oral contraceptives	
LFUI		Long Form Unique Identifier		Identifies a unique long form		L121977	
NormLF		Normalized Long Form		Lexically normalized version of the long form		oral contraceptive	
Source		Source Inventory		Name of the source sense inventory		ADAM	
Modified		Modified		Modified by quality control or not		modified	
Auxiliary*							
**Data Field**	**Name**		**Description**		**Source**		**Example**
SFEUI	Short Form Entry Unique Identifier		Identifies a unique UMLS short form		UMLS-LRABR		E0319213
LFEUI	Long Form Entry Unique Identifier		Identifies a unique UMLS long form		UMLS-LRABR		E0044077
Type	Type of Entry		Abbreviation or acronym		UMLS-LRABR		acronym
PrefSF	Preferred Short Form		Preferred version of a short form		ADAM		o.c.
Count	Count		Number of occurrences in the corpus		ADAM, Vanderbilt		10
Score	Score		Adjusted proportion of occurrences		ADAM		0.7357
Frequency	Frequency		Frequency of the sense in the corpus		Vanderbilt		0.4168
UMLS.CUI	UMLS Concept Unique Identifier		UMLS CUI that mapped to the sense		Vanderbilt		c0009905

*Auxiliary data fields are unique to a single source and found only in the “auxiliary” version of the Meta-Inventory available in the GitHub repository (https://bit.ly/github-clinical-abbreviations). Abbreviations: UMLS, Unified Medical Language System; LRABR, Lexical Resource for Abbreviations and Acronyms; ADAM, Another Database of Abbreviations in Medline.

The Meta-Inventory contains 405,543 unique records (i.e., rows or source entries), increasing by 40% the unique records available in the major repository (UMLS-LRABR). Out of the 405,543 total records, only 107,650 (27%) do not have any synonymous records. This highlights the important role of cross-mapping to reduce redundancy. The Meta-Inventory represents 104,057 unique abbreviations (i.e., short forms) and 373,930 unique pairs, increasing by 45% the unique abbreviations and 28% the unique pairs available in the major repository (UMLS-LRABR). This highlights the benefit of augmenting the major repository (UMLS-LRABR) with clinically-oriented inventories, which contain more clinically-oriented and therefore unique abbreviations and pairs.

The Meta-Inventory represents 170,426 unique senses (i.e., long forms) and 183,817 unique groups. On average, each abbreviation has 1.77 (range: 1–142) possible senses after cross-mapping. Importantly, 24,090 abbreviations (23%) had more than one sense, and 7,113 abbreviations (7%) had four or more senses. The abbreviation “PA” had the most possible senses (142), including pancreatic adenocarcinoma, physician assistant, Pennsylvania, arterial pressure, psoriatic arthritis, pseudomonas aeruginosa, and many others. This highlights the difficulty of disambiguating abbreviations in clinical NLP, as opposed to words, which have at most three or four possible senses.

## Technical Validation

### Quality control

Errors have been recognized in several source inventories^20,39^. To address this problem and achieve a reliable database, we implemented a semi-automated quality control process to identify, then modify or retire, erroneous records. We chose to modify rather than retire where possible to maintain completeness^47,48^. Four rule-based heuristics were used to automatically identify potential errors, including [1] exact duplicates within the same source, [2] records with excessive or misplaced punctuation (e.g., “..MS”), [3] records where alphanumeric characters in the short form did not occur in the long form, and [4] records with spelling errors. To identify spelling errors, each long form was compared against a medical word corpus derived from the UMLS Metathesaurus using a Python-based spell checker (https://pypi.org/project/pyspellchecker/). After potential errors were flagged by heuristics, a clinician manually verified each flagged record as erroneous or not. Duplicate records were retired to a separate database. Non-duplicate records (i.e., those with excess punctuation, missing characters, or spelling errors) were either corrected by the clinician, or retired if correction was not possible. 4312 records were corrected or retired. Corrected records were marked as “modified” in a separate data field. Documentation of the changes and copies of the original records can be found in the repository.

### Cross-mapping validation

To validate the cross-mapping, two clinicians independently reviewed a random 5% subsample of synonymous groups with two or more records from difference sources (~2,000 synonymous groups). The clinicians evaluated non-ambiguity (at most one meaning per group)^47,48^. Inter-rater reliability was good [agreement = 99.8%; Cohen’s kappa = 0.71], and disagreements were resolved by discussion. The clinicians found 99.49% of groups non-ambiguous. This highlights the reliability of the cross-mapping method and suggests the error rate of cross-mapping is extremely low (less than 0.2%).

Additionally, two clinicians independently reviewed a random 0.5% subsample of short forms with five or more records (~100 short forms). The clinicians evaluated the percentage of groups which could have been grouped further (i.e., failure to remove redundancy). Inter-rater reliability was good [agreement = 92%; Cohen’s kappa = 0.84], and disagreements were resolved by discussion. The clinicians found that only 11% of groups could have been grouped further. This suggests that cross-mapping resolved most of the redundancy in the Meta-Inventory.

### Coverage evaluation

An important reason why we created the Meta-Inventory was to improve completeness, or *coverage* of every abbreviation and its senses in clinical text. Evaluating coverage is critical to determine whether the Meta-Inventory achieved this goal. To evaluate coverage in clinical text, we used MIMIC-III, a publicly-available corpus of over 2 million de-identified critical care notes at Beth Israel Deaconess Medical Center^55^. MIMIC-III is ideal because: [1] it is unrelated to any corpora used to generate the sources, and [2] it is from a different geographic region and medical specialty than the sources. Therefore, MIMIC-III allowed us to evaluate coverage on completely new and distinct corpus of clinical texts.

**Fig. 2:**
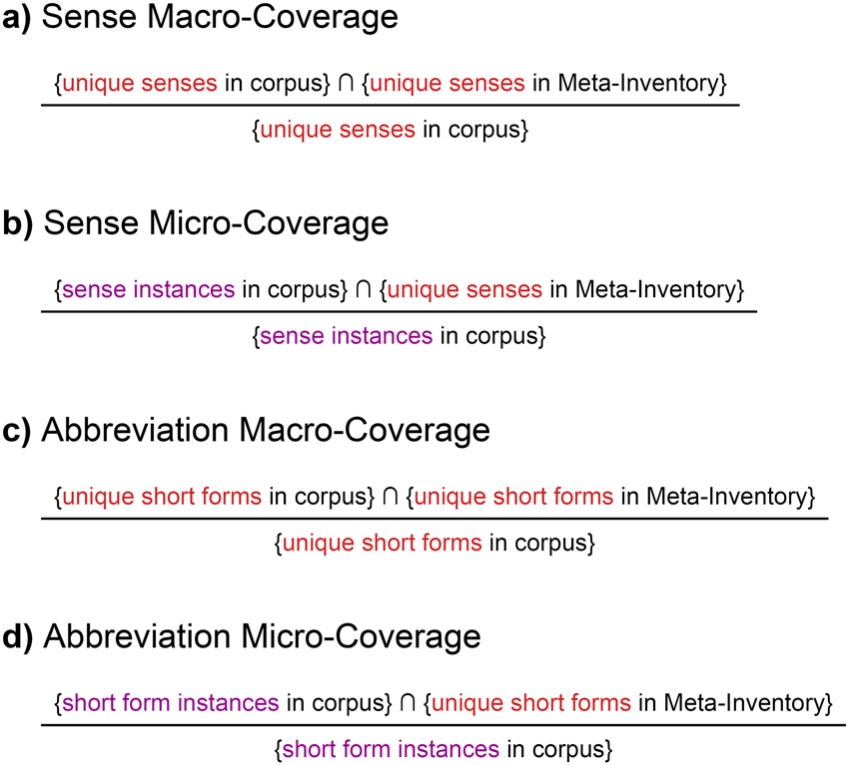
Formulas for Calculating Coverage.

We calculated coverage of both abbreviations (*abbreviation coverage*) and their senses (*sense coverage*). For each, we calculated *macro-coverage*, which computes the metric for each abbreviation or sense and then averages them, as well as *micro-coverage*, which treats every instance independently. Figure 2 displays formulas for computing all four metrics. To identify abbreviations in MIMIC-III, we used the previously-mention CARD framework. A clinician manually reviewed the CARD-identified abbreviations to remove obvious errors (i.e., non-abbreviations) such as misspellings like “folllowed” or aggregated words like “lip/chin.” To identify senses in MIMIC-III, a clinician manually annotated 60 randomly-selected instances of 60 randomly-selected abbreviations with multiple senses.

**Fig. 3:**
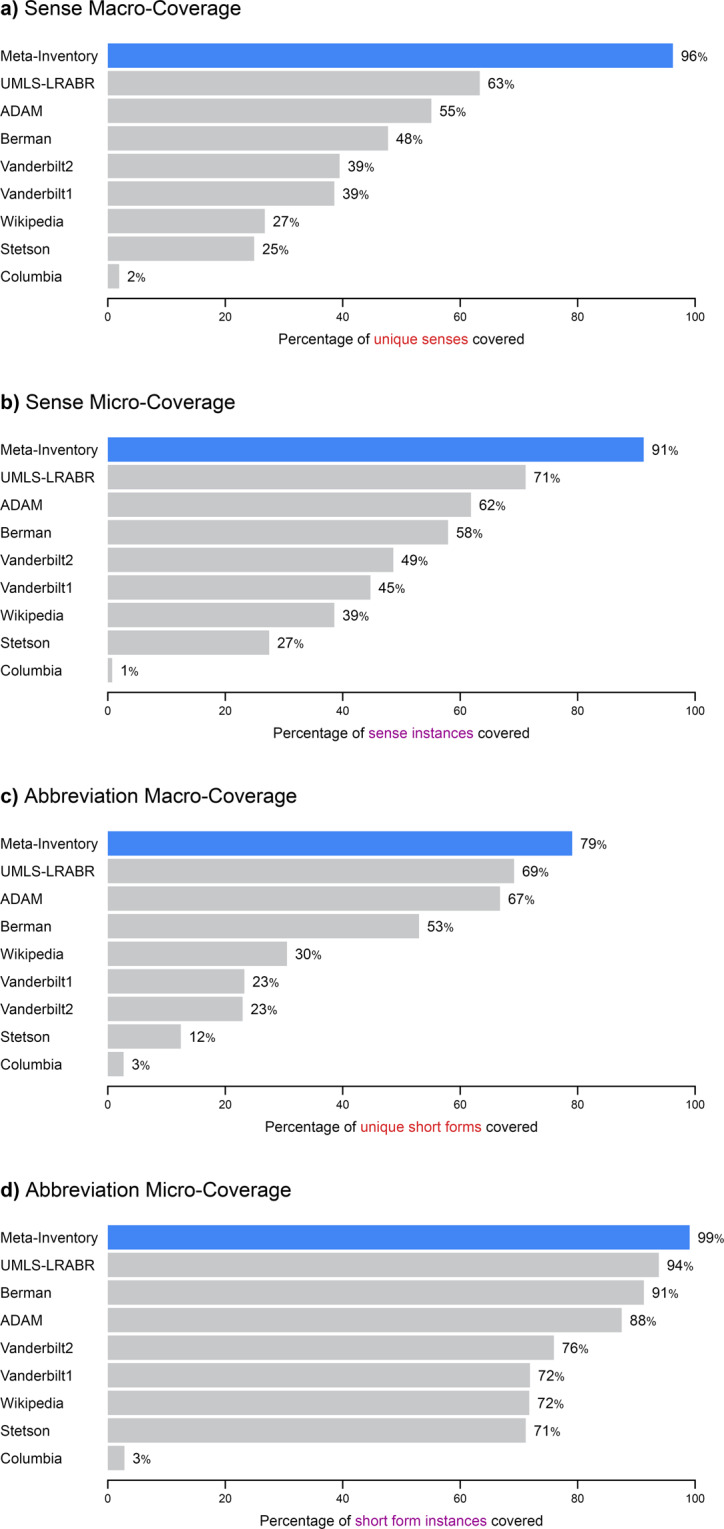
Coverage Estimates for the Meta-Inventory and its Sources.

Figure 3 displays coverage estimates for the Meta-Inventory compared with its individual sources. The Meta-Inventory had high coverage, with a sense macro-coverage of 96%, sense micro-coverage of 91%, abbreviation macro-coverage of 79%, and abbreviation micro-coverage of 99%. This represents a substantial increase in sense coverage (28% to 52%) and abbreviation coverage (6% to 14%) over the major repository (UMLS-LRABR). This suggests that the Meta-Inventory is sufficiently comprehensive to recognize almost every abbreviation and its senses in a given clinical text in the United States.

## Usage Notes

The Meta-Inventory is the most complete compilation of medical abbreviations and acronyms in American English. It includes records from varied corpora, medical specialties, and geographic regions, which is necessary to support interoperability (i.e., cross- or multi-institutional recognition, disambiguation, and expansion of abbreviations). The Meta-Inventory’s completeness is notable because it can be applied to a diversity of clinical texts, not only specialty- or institution-specific ones. In addition to being comprehensive, the Meta-Inventory is quality-controlled and uses state-of-the-art machine learning methods to automatically reduce redundancy. Application of machine learning to data engineering improves speed and scale^56^, and our approach could be applied to similar problems with data harmonization, integration, and cross-mapping in the future.

Cross-mapping is critical to ensure *concept-orientedness*, a known requirement of controlled vocabularies such as the Meta-Inventory^47,48^. Concept-orientedness states that records “must correspond to at least one meaning (non-vagueness) and no more than one meaning (non-ambiguity), and that meanings correspond to no more than one record (non-redundancy).” Concept-orientedness is important to enhance interpretability by human users, and may improve processing speeds of downstream tasks. For example, in the Meta-Inventory, using group rather than record identifiers for recognizing abbreviations could reduce linear processing time by 55%, since the Meta-Inventory contains 405,543 records but only 183,817 groups. This might impact processing of extremely large amounts of text. In this way, the Meta-Inventory maximizes comprehensiveness while minimizing the potential negative impacts of redundancy and large size.

While every effort has been made to increase completeness and reduce redundancy, some limitations must be acknowledged. *First*, the Meta-Inventory does not yet contain abbreviations from every medical specialty and potential setting, which may limit its completeness in certain contexts. However, we envision that institutions could easily extend the Meta-Inventory using their own corpora and the process we have reported on. *Second*, some unresolved redundancy is present in the Meta-Inventory. An extremely high-specificity threshold was used when cross-mapping. This prevented any inaccurate cross-mapping, as intended, but may have also prevented accurate cross-mapping to some degree. We believe this was an acceptable trade-off to ensure complete confidence in the cross-mapping we did perform, even though it meant that some redundancy remained.

To mitigate these limitations, we encourage users of the Meta-Inventory to participate in its improvement and maintenance. Please email the corresponding author or, preferably, submit a request via the GitHub repository. We welcome and greatly appreciate any efforts, including but not limited to: [1] identification of potential additional sources, and [2] reports of unresolved errors or redundancy. We anticipate that the Meta-Inventory will continue to be updated, as new literature gets published, new inventories are made, errors are identified, and redundancy is removed.

As an important and final observation, the Meta-Inventory, although needed, cannot solve the challenge of abbreviations in clinical NLP alone. Recognition, disambiguation, and expansion of abbreviations is complicated by misspellings (e.g., LEVF vs. LVEF), variation (e.g., EtOH vs. ETOH), plurals (e.g., MRI vs. MRIs), inflection (e.g., D/C vs. D/C’ed), and other challenges^13,57^ which the Meta-Inventory does not address. Additional research is needed to improve methods that normalize and disambiguate abbreviations, which will support better clinical NLP in combination with the Meta-Inventory.

## Data Availability

We used the Python programming language for all activities. The entire code is permanently available in Zenodo (https://zenodo.org/record/4266962)^54^ or GitHub (https://bit.ly/github-clinical-abbreviations).

## References

[1] Yim WW, Yetisgen M, Harris WP, Sharon WK (2016). Natural Language Processing in Oncology: A Review. JAMA Oncol..

[2] Pons E, Braun LMM, Hunink MGM, Kors JA (2016). Natural language processing in radiology: A systematic review. Radiology.

[3] Kreimeyer K (2017). Natural language processing systems for capturing and standardizing unstructured clinical information: A systematic review. J. Biomed. Inform..

[4] Demner-Fushman D, Chapman WW, McDonald CJ (2009). What can natural language processing do for clinical decision support?. J. Biomed. Inform..

[5] Miller DD, Brown EW (2018). Artificial Intelligence in Medical Practice: The Question to the Answer?. Am. J. Med..

[6] Murff HJ (2011). Automated identification of postoperative complications within an electronic medical record using natural language processing. JAMA - J. Am. Med. Assoc..

[7] Devlin, J., Chang, M.-W., Lee, K. & Toutanova, K. BERT: Pre-training of Deep Bidirectional Transformers for Language Understanding. *arXiv* (2019).

[8] Peng, Y., Yan, S. & Lu, Z. Transfer Learning in Biomedical Natural Language Processing: An Evaluation of BERT and ELMo on Ten Benchmarking Datasets. *arXiv*10.18653/v1/w19-5006 (2019).

[9] Nayak, P. Google product updates: Understanding searches better than ever before. *The Keyword: The Official Google Blog* https://www.blog.google/products/search/search-language-understanding-bert/ (2019).

[10] Garg, S., Vu, T. & Moschitti, A. TANDA: Transfer and Adapt Pre-Trained Transformer Models for Answer Sentence Selection. *arxiv* (2019).

[11] Shickel B, Tighe PJ, Bihorac A, Rashidi P (2018). Deep EHR: A Survey of Recent Advances in Deep Learning Techniques for Electronic Health Record (EHR) Analysis. IEEE J. Biomed. Heal. Informatics.

[12] Jiang M (2011). A study of machine-learning-based approaches to extract clinical entities and their assertions from discharge summaries. J. Am. Med. Informatics Assoc..

[13] Moon S, McInnes B, Melton GB (2015). Challenges and practical approaches with word sense disambiguation of acronyms and abbreviations in the clinical domain. Healthc. Inform. Res..

[14] Jimeno-Yepes AJ, McInnes BT, Aronson AR (2011). Exploiting MeSH indexing in MEDLINE to generate a data set for word sense disambiguation. BMC Bioinformatics.

[15] Pesaranghader A, Matwin S, Sokolova M, Pesaranghader A (2019). DeepBioWSD: Effective deep neural word sense disambiguation of biomedical text data. J. Am. Med. Informatics Assoc..

[16] Jin Q, Liu J, Lu X (2019). Deep Contextualized Biomedical Abbreviation Expansion. arXiv.

[17] Wu, Y., Xu, J., Zhang, Y. & Xu, H. Clinical Abbreviation Disambiguation Using Neural Word Embeddings. *Proc. 2015 Work. Biomed. Nat. Lang. Process*. 10.18653/v1/w15-3822 (2015).

[18] Li, I. *et al*. A Neural Topic-Attention Model for Medical Term Abbreviation Disambiguation. 1–9 (2019).

[19] Wu Y (2012). A comparative study of current Clinical Natural Language Processing systems on handling abbreviations in discharge summaries. AMIA Annu. Symp. Proc..

[20] Grossman LV, Mitchell EG, Hripcsak G, Weng C, Vawdrey K (2018). A Method for Harmonization of Clinical Abbreviation and Acronym Sense Inventories. J. Biomed. Inform..

[21] Ehrmann, M., Della Rocca, L., Steinberger, R. & Tannev, H. Acronym recognition and processing in 22 languages. *Int. Conf. Recent Adv. Nat. Lang. Process. RANLP* 237–244 (2013).

[22] The Joint Commission. Standard MOI.4: Use of Codes, Symbols, and Abbreviations. https://www.jointcommissioninternational.org/en/standards/hospital-standards-communication-center/use-of-codes-symbols-and-abbreviations/ (2020).

[23] Awan S (2016). Use of medical abbreviations and acronyms: Knowledge among medical students and postgraduates. Postgrad. Med. J..

[24] Chemali M, Hibbert EJ, Sheen A (2015). General practitioner understanding of abbreviations used in hospital discharge letters. Med. J. Aust..

[25] Hamiel U (2018). Frequency, comprehension and attitudes of physicians towards abbreviations in the medical record. Postgrad. Med. J..

[26] Shilo L, Shilo G (2018). Analysis of abbreviations used by residents in admission notes and discharge summaries. QJM An Int. J. Med..

[27] Liu H, Lussier YA, Friedman C (2001). A study of abbreviations in the UMLS. AMIA Symp. Annu. Proc..

[28] Xu H, Stetson PD, Friedman C (2007). A study of abbreviations in clinical notes. AMIA Annu. Symp. Proc..

[29] Savova GK (2008). Word sense disambiguation across two domains: Biomedical literature and clinical notes. J. Biomed. Inform..

[30] Wu Y (2017). A long journey to short abbreviations: developing an open-source framework for clinical abbreviation recognition and disambiguation (CARD). J. Am. Med. Inform. Assoc..

[31] Xu H, Stetson PD, Friedman C (2009). Methods for Building Sense Inventories of Abbreviations in Clinical Notes. J. Am. Med. Informatics Assoc..

[32] Moon S, Pakhomov S, Liu N, Ryan JO, Melton GB (2014). A sense inventory for clinical abbreviations and acronyms created using clinical notes and medical dictionary resources. J. Am. Med. Informatics Assoc..

[33] Dannélls, D. *Automatic acronym recognition*. *Proceedings of the Eleventh Conference of the European Chapter of the Association for Computational Linguistics: Posters & Demonstrations on - EACL* ’*06*10.3115/1608974.1608999 (2006).

[34] MetaMap - A Tool For Recognizing UMLS Concepts in Text. https://metamap.nlm.nih.gov/ (2016).

[35] Wu Y (2015). A Preliminary Study of Clinical Abbreviation Disambiguation in Real Time. Appl. Clin. Inform..

[36] Wu, Y. *et al*. Clinical acronym/abbreviation normalization using a hybrid approach. *CEUR Workshop Proc*. **1179** (2013).

[37] Szczuka, M. & Ślȩzak, D. How deep data becomes big data. *Proc. 2013 Jt. IFSA World Congr. NAFIPS Annu. Meet. IFSA/NAFIPS 2013*10.1109/IFSA-NAFIPS.2013.6608465 (2013).

[38] Chen Z (2018). Understand what happened under the surface: Tracing dynamic deep data. Proc. - 2017 Int. Conf. Inf. Syst. Comput. Sci. INCISCOS 2017.

[39] Cimino JJ (1998). Auditing the Unified Medical Language System with Semantic Methods. J. Am. Med. Informatics Assoc..

[40] UMLS Reference Manual. https://www.ncbi.nlm.nih.gov/books/NBK9680/ (2016).

[41] Zhou W, Torvik VI, Smalheiser NR (2006). ADAM: Another database of abbreviations in MEDLINE. Bioinformatics.

[42] Berman JJ (2004). Pathology Abbreviated: A Long Review of Short Terms. Arch. Pathol. Lab. Med..

[43] Wikipedia: List of Medical Abbreviations. https://en.wikipedia.org/wiki/List_of_medical_abbreviations (2016).

[44] Recognition and Disambiguation of Clinical Abbreviations. https://sbmi.uth.edu/ccb/resources/abbreviation.htm (2016).

[45] Stetson PD, Johnson SB, Scotch M, Hripcsak G (2002). The sublanguage of cross-coverage. AMIA Annu. Symp. Proc..

[46] Bodenreider O (2004). The Unified Medical Language System (UMLS): integrating biomedical terminology. Nucleic Acids Res..

[47] Cimino JJ (1998). Desiderata for controlled medical vocabularies in the twenty-first century. Methods Inf. Med..

[48] Cimino JJ (2006). In defense of the Desiderata. J. Biomed. Inform..

[49] Hole WT (2004). Achieving ‘source transparency’ in the UMLS Metathesaurus. Stud. Health Technol. Inform..

[50] Lu CJ, Payne A, Mork JG (2020). The Unified Medical Language System SPECIALIST Lexicon and Lexical Tools: Development and applications. J. Am. Med. Informatics Assoc..

[51] Wang, Y. *et al*. MedSTS: A resource for clinical semantic textual similarity. *arXiv* (2018).

[52] Ke G (2017). LightGBM: A highly efficient gradient boosting decision tree. Adv. Neural Inf. Process. Syst..

[53] Huang, K., Altosaar, J. & Ranganath, R. ClinicalBERT: Modeling Clinical Notes and Predicting Hospital Readmission. *arXiv* (2019).

[54] Grossman Liu L (2021). Zenodo.

[55] Johnson, A. E. W. *et al*. MIMIC-III, a freely accessible critical care database. *Sci. Data* **3** (2016). 10.1038/sdata.2016.35 PMC4878278 27219127

[56] Ratner A (2017). Snorkel: Rapid training data creation with weak supervision. Proc. VLDB Endow..

[57] Nadkarni PM, Ohno-Machado L, Chapman WW (2011). Natural language processing: An introduction. J. Am. Med. Informatics Assoc..

